# Investigation of the reversibility of freeze/thaw stress-induced protein instability using heat cycling as a function of different cryoprotectants

**DOI:** 10.1007/s00449-020-02327-3

**Published:** 2020-03-20

**Authors:** Anna K. Wöll, Jürgen Hubbuch

**Affiliations:** Institute of Engineering in Life Sciences, Section IV: Biomolecular Separation Engineering, Karlsruhe Institute of Technology (KIT), Fritz-Haber-Weg 2, 76131 Karlsruhe, Germany

**Keywords:** Aggregation, Heat-induced reversibility, Phase diagram, Morphology, Lysozyme

## Abstract

**Abstract:**

Formulation conditions have a significant influence on the degree of freeze/thaw (FT) stress-induced protein instabilities. Adding cryoprotectants might stabilize the induced FT stress instabilities. However, a simple preservation of protein stability might be insufficient and further methods are necessary. This study aims to evaluate the addition of a heat cycle following FT application as a function of different cryoprotectants with lysozyme as exemplary protein. Sucrose and glycerol were shown to be the most effective cryoprotectants when compared to PEG200 and Tween20. In terms of heat-induced reversibility of aggregates, glycerol showed the best performance followed by sucrose, NaCl and Tween20 systems. The analysis was performed using a novel approach to visualize complex interplays by a clustering and data reduction scheme. In addition, solubility and structural integrity were measured and confirmed the obtained results.

**Graphic abstract:**

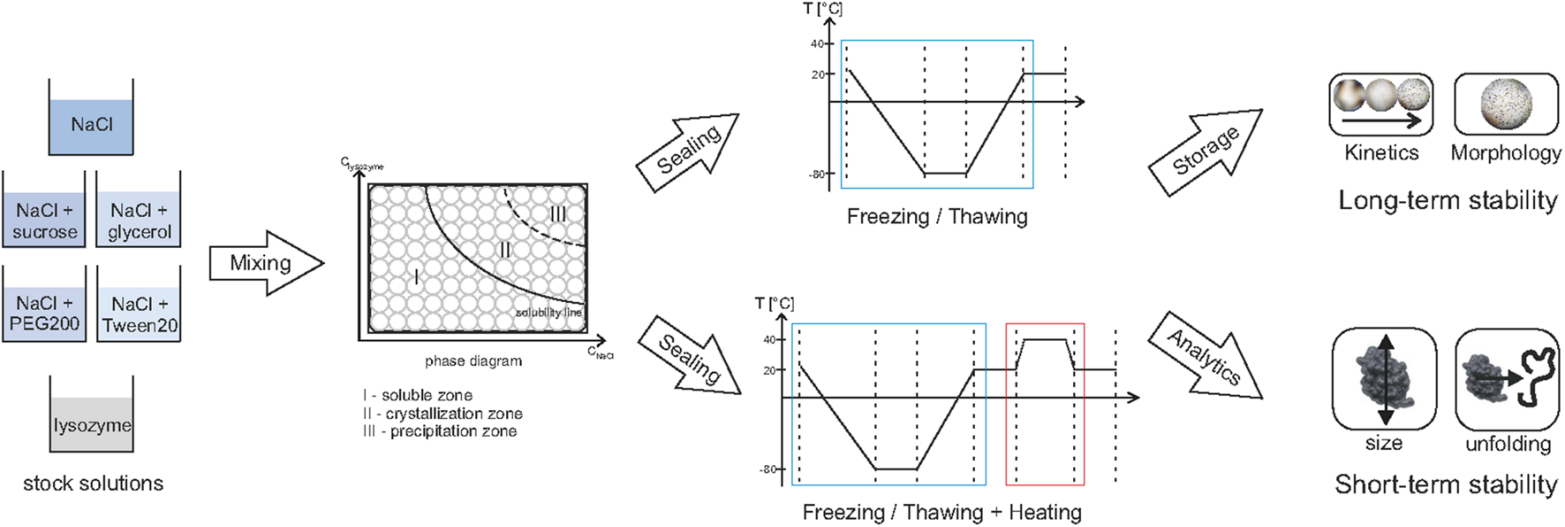

**Electronic supplementary material:**

The online version of this article (10.1007/s00449-020-02327-3) contains supplementary material, which is available to authorized users.

## Introduction

- Freezing and thawing enable a higher degree of flexibility during manufacturing and improve long-term stability during storage [1–3]. To guarantee protein stability, chemically, mechanically, and physically induced stresses, including cold temperatures, have to be taken into account [4–7]. The influence of freezing and thawing on the protein stability is complex and depends on formulation parameters (buffer, excipient, and protein type and concentration, pH value), system parameters (freezing and thawing point, glass transition and clouding point), and process parameters (cycle number, freezing/thawing ramp, storage temperature and time) [4, 7–14]. The induced FT stress types might cause protein damage, which implies colloidal instabilities (aggregate formation), conformational instabilities (structural changes), and biological activity loss [3–5, 15, 16]. The different FT stress types include several processes. Freeze concentration of all solutes (e.g. buffer components, excipients, protein) [11, 17] due to ice crystal formation. Furthermore, liquid–liquid phase separation (LLPS) might take place, which can be due to the increasing concentrations [17], and/or the decreasing temperature, also called cloud point (T_cloud_) [9, 18]. The decreasing temperature might result in cold denaturation, which is the structural loss of proteins with quaternary structure [19]. An additional issue is the growing ice surface, due to the possible denaturation of the protein molecules on this surface [7, 20]. Consequently, protein aggregates, native or non–native, might occur because of the mentioned effects. Protein aggregates can appear through different mechanisms [21], depending on the protein surface charge, conformational changes, and excipients in the solutions. These parameters influence intermolecular and intramolecular interactions of proteins and/or excipients. On the one hand, when covalent binding (e.g. disulfide bonds) arises, aggregates are irreversibly bonded to each other. On the other hand, when non–covalent binding (e.g. electrostatic, hydrophobic, van der Waals [22, 23]) occurs, aggregates might be reversible [21, 24–27]. Normally, in order to stabilize proteins in solution and to prevent aggregation, the formulation is adjusted using different excipients. To inhibit FT-induced instabilities, cryoprotectants are used [4, 11, 28]. The excipients, including cryoprotectants, show different mechanisms to stabilize or destabilize proteins in solution. The excipients are either preferentially included or excluded from the proximity environment, whereas preferential exclusion, or hydration, results in hydration of the protein surface which induces a stabilizing effect [29, 30]. As a function of the type and strength of the resulting interactions, protein aggregates form and can be classified irreversible or reversible. Of special interest are reversible native aggregates. Among these, one has to differentiate between two kinds of reversible aggregates: (a) aggregates are in equilibrium with monomers, and (b) induced aggregation by perturbation of the solution conditions (pH, temperature, etc.). The first type of aggregates (a) is reversible by diluting the solution, whereas for the second type of aggregates, (b) original solution conditions need to be re-established. Additionally, it was shown to be possible to dissociate reversible protein aggregates by special treatments, for example through the application of heat [26]. This said, heat might cause protein denaturation and/or induce irreversible aggregation [21]. Taken all together, an investigation of the reversibility/dissociability of FT stress-induced protein aggregates as a function of different excipients using heat cycling is currently missing and promises new insights when dealing with reversible aggregation during bioprocessing.

In this study the influence of different excipients, known to be cryoprotectants, on FT stress-induced colloidal instability on the long-term stability of lysozyme is investigated, whereas colloidal instability is defined as a change in the phase behavior and/or crystal morphology. The excipients chosen belong to different groups, namely two osmolytes (the sugar sucrose and the polyol glycerol), a polymer (polyethylene glycol 200 (PEG200)), and a surfactant [Polysorbate 20 (Tween20)]. Subsequently, the reversibility of these induced instabilities by including a heat cycle to the respective FT protocol is investigated. In order to visualize the occurrences, phase diagrams were chosen and systems clustered using a MPPD approach. The descriptors for clustering chosen consisted out of morphological and rate values. Additionally, solubility and protein structure of the different systems were measured.

## Material and methods

In this study, phase behavior, aggregation kinetics, and morphology of aggregates were investigated by creating a multidimensional protein phase diagram (MPPD). In addition, the solubility line (SL) was calculated and the colloidal stability (size) as well as the conformational stability (protein structure) was studied. In the following, the preparation of the stock solutions, the creation of the MPPD, the performance of the FT-cycling with and without an additional heat step, as well as the analytical methods used (dynamic light scattering (DLS) and Fourier–transform infrared spectroscopy (FTIR)) are explained.

### Preparation of stock solutions

For each experiment, three buffers (base buffer, salt buffer, excipient buffers) and a protein stock solution had to be prepared. As base buffer, a - mM multi-component buffer (MCB) was used. It was created using a tool described by Kröner et al*.* [31] and consisted of the buffer substances AMPSO (Sigma–Aldrich), TAPSO (Sigma–Aldrich), MES (AppliChem GmbH, Darmstadt, Germany), formic acid (Merck KGaA), and D-(+)-malic acid (Sigma–Aldrich). Furthermore, the pH value was adjusted, using NaOH or HCl (Merck KGaA), with a five-point calibrated pH meter (HI-3220, Hanna® Instruments, Woonsocket, RI, USA) equipped with a SenTix^®^ 62 pH electrode (Xylem Inc., White Plains, NY, USA). The pH value was adjusted to a value differing by up to ± 0.1 pH units from the final pH value. Prior to the use of the buffer, the pH value was verified again and finally adjusted to a value differing by only ± 0.02 pH units. In addition, the ionic strength (IS) was adjusted to 10.08 ms/cm with an accuracy of ± 1 ms/cm at 24 °C ± 1 °C using the conductivity meter CDM 230 (Radiometer Analyticals, Lyon, France) and the four-point calibrated conductivity cell E61M014 (Radiometer Analyticals, Lyon, France) using NaCl (Merck KGaA). A salt buffer with different concentrations, 2.0 M, 3.75 M, and 4.29 M, was prepared. The respective amount of NaCl (Merck KGaA) was dissolved in the base buffer. The same procedure was performed on the excipient buffer. Stock excipient buffer with 1.8 M sucrose (Sigma-Aldrich), 3 M glycerol (VWR, Radnor, PA, USA), 84 mM polyethylene glycol 200 (PEG200)(Sigma-Aldrich), and 0.72 mM polysorbate 20 (Tween20) (AppliChem GmbH) were prepared. The pH values of all additives (salt, and excipient) were adjusted on the day of preparation and prior to use as described above. All buffers were filtered through a 0.2-µm Supor^®^ Polyethersulfone (Pall Corporation, Port Washington, NY, USA) filter and stored at room temperature. The buffers were not used for longer than 2 weeks after preparation. The protein stock solution was prepared with lyophilized lysozyme from chicken egg white (Hampton Research, Aliso Viejo, CA, USA).

The protein was dissolved in the base buffer and filtered through a 0.2-µm syringe cellulose acetate filter (VWR). A desalting step was attached to remove aggregates and production-related additives, using PD-10 (GE Healthcare, Uppsala, Sweden) columns and the respective spin protocol [32]. To adjust the final protein concentration of 87 mg/mL, a 1:10 dilution was prepared and measured using the NanoDrop™ 2000c UV–Vis spectrophotometer (Thermo Fisher Scientific, Waltham, MA, USA). Thereby, an extinction coefficient of E^1% ^(280 nm) = 22.00 Lg/cm was used for the measurement. The protein stock solution was prepared freshly and was not used for more than one day.

### Phase diagrams

To create the phase diagrams, a method described by Baumgartner et al*.* was used [33]. The final lysozyme concentrations varied between 2.5 mg/mL and 25 mg/mL, and the final NaCl concentrations between 0 M and 2.5 M. The excipient concentration was kept constant at 300 mM sucrose, 1000 mM glycerol, 6.8 mM PEG200 or 0.03 mM Tween20. The protein and salt stock solutions were placed onto a Freedom EVO® 100 fully automated liquid handling station (Tecan Group Ltd., Männedorf, Switzerland) platform. The liquid handling station is equipped with fixed tips and 250 µL dilutors and controlled by Freedom EVO^®^ 2.4 SP3 (Tecan Group Ltd.). The protein and salt concentration dilution rows were prepared in a Deepwell PP plate (Greiner Bio-one, Frickenhausen, Germany). After the salt dilution row was prepared, either 57.2 µL of sucrose or glycerol or 25 µL of PEG200 or Tween20 was added and mixed manually. Then, the phase diagram was created automatically in a MRC Under Oil 96–well Crystallization Plate (SWISSCI AG, Neuheim, Switzerland), whereas 18 μL of the salt/additive solution was mixed with 6 μL protein solution. Before the plates were sealed with Duck^®^ Brand HD Clear sealing tape (ShurTech^®^ brands, Avon, OH, USA), to avoid evaporation, plates were centrifuged in an Eppendorf centrifuge 5810 R (Eppendorf AG, 100 Hamburg, Germany) at 1000 rpm for 1 min to remove all air bubbles. After performing the FT protocols with and without heat cycling (“Cycling”), the plates were placed in the incubation chamber RockImager 54 (Formulatrix, Bedford, MA, USA) at 20 °C for 40 days.

### Cycling

#### FT cycling

FT protocol with different cycles numbers (FT cx; *x* = 0, 1, 3) were carried out. A plate at FT c0, not subjected to any FT stress application, was used as a reference plate. This plate was directly placed into the incubation chamber after preparation. The other plates were placed onto the cryogenic device EF600M 105 (Grant Instruments, Cambridgeshire, UK) after preparation. The plate handling and the adjustments on the cryogenic device are described in the publication by Wöll et al. [13]. In this study, all plates were frozen at 0.5 ℃/min and thawed at 2.5 ℃/min.

#### Heat cycling

Heat cycling following FT cycling was performed (FT cy h; y = 1, 3) as follows: The respective plates were heated to 40 ℃ for 30 min using a HLC Cooling-ThermoMixer MKR 13 (Ditabis AG, Pforzheim, Germany). Upon completion, the plates were directly placed in the incubation chamber at 20 ℃.

### Multidimensional protein phase diagrams (MPPD)

To evaluate phase transitions, long-term stability and reversibility of aggregates of the used lysozyme formulation, an MPPD was employed [33]. An MPPD combines data on morphology, kinetic and aggregation obtained from images taken during storage, in one figure by data reduction and subsequent clustering. In this study, the following data were extracted manually from the images: (1, 2) length and width of a maximum of eight crystals in µm, (3) percentage aggregation amount per well (*n*_Agg_), (4) aggregation onset time (*t*_onset_) in hours, and aggregation growth time (*t*_G_) in hours. The mean crystal 120 length (*L*_*C*_) and width (*W*_*C*_) as well as the interquartile range (IQR) of the crystal lengths (∆*L*_*C*_) and widths (∆*W*_*C*_) were calculated. In addition, the ratio of the length and width (*L*_*C*_: *W*_*C*_) and the interquartile range of this ratio [∆(*L*_*C*_: *W*_*C*_)] were calculated. Using all of these mentioned parameters, the MPPDs were constructed. The used settings for the MPPD construction have been described in a previous work [34], where the clustering algorithm selected an optimal cluster number between three and ten clusters.

In addition, to evaluate the occurrence of each cluster, the total amount of each cluster per phase diagram was determined and the occurrence in percentage per plate was calculated.

### Analytics

In order to evaluate if structural parameter alter due to (a) initial stress and (b) heat reversibility, FTIR and DLS measurements were applied. Stable conditions were chosen at 18 mg/mL and 22 mg/mL at the four lowest salt concentrations (0.00, 0.23, 0.45, and 0.68 M). A condition was only analyzed when no visible aggregation appeared prior to the actual measurement (*t*_onset_ > *t*_0_). This resulted in a total amount of 151 samples (non-stressed (FT c0), stressed (FT c1 and FT c3), stressed and heated (FT c1 h and FT c3 h). In addition, supernatant measurements of all phase diagrams were performed to calculate SLs. The protein stock solution for the analytics was prepared as described in “Preparation of stock solutions”. The pipetting was performed manually, whereas the protein, salt, and excipient solutions were mixed in the same ratio as was done for the phase diagrams, see “Phase diagrams”. A maximum of 180 µL of the samples was prepared in 0.5 mL Eppendorf tubes (Eppendorf AG). For the stressed samples (FT cx; *x* = 1, 3 and FT-heating FT cy h; *y* = 1, 3), the samples were split into up to six 30 µL proportions and pipetted in the crystallization plate, after preparation in 0.5 mL Eppendorf tubes (Eppendorf AG). Afterwards, plate handling was done as described in “Phase diagrams” and “Cycling”. After the respective stress protocol was performed, the samples were pipetted back into Eppendorf tubes and mixed. Before the analytical method could be performed, the samples were filtered, using an Eppendorf centrifuge 5810 R (Eppendorf AG) at 2000 g for 5 min, through a 0.2-µm AcroPrep™ 96 filter plate (350 µL)(Pall Corporation, New York, New York, USA) into a 96–well PP-Microplate (U-shape)(Greiner Bio-one). After filtration, the samples were split to be used with the different analytical methods which are described below.

#### Dynamic light scattering (DLS)

The hydrodynamic radius of the protein in the respective solution was measured with DLS using the Wyatt DynaPro Plate Reader I (Wyatt,Santa Barbara, California, USA) and a polystyrene 384-well assay plate (Corning Inc., Corning, New York, USA). Therefore, 25 µL of the filtered sample was pipetted into the wells in triplicate and was covered with 10 µL Xiameter™ PMX-200 Silicon fluid 20cs (Dow Corning Inc. Midland, Michigan, USA) to avoid evaporation. The plate was centrifuged in an Eppendorf centrifuge 5810 R (Eppendorf AG) at 400 g for 1 min to remove all air bubbles. Next, the plate was placed in the plate reader and each sample was measured twice at 20 ℃ with an acquisition time of 5 s and an acquisition number of 10 as well as automatic attenuation.

#### Fourier-transform infrared spectroscopy (FTIR)

To investigate changes in the secondary protein structure FTIR spectroscopy, a Tensor 27 (Bruker Optics, Ettlingen, Germany) was used. The FTIR was equipped with a cryo-cooled mercury cadium telluride (CC-MTC) narrow detector (Bruker Optics) and a BioATR II crystal (Bruker Optics) and controlled by OPUS 7.2 (Bruker Optics). For the measurement, 25 µL of background or sample was pipetted onto the crystals, covered with a lid, and then measured for 5 min (mirror speed of 160 kHz) with a resolution of 2 cm^−1^ in a range from 3500 to 900 cm^−1^. The background subtraction, as well as automatic compensation, was automatically performed by the software. Additionally, data pre-processing was performed. After atmospheric compensation and vector normalization, the data were smoothed using a Savitzky–Golay filter with a second-order polynomial and a frame length of 17 in a wavenumber region from 1750 to 1550 cm^−1^. This data were then used to calculate the average of the samples measured in duplicate. The area within the amid I range (1600–1700 cm^−1^) for the α-helix, β-sheet, and β-sheet antiparallel was extracted using the *trapz* function available in MATLAB (Version 2019b). Therefore, the peak minimum for α-helix (1650–1685 cm^−1^), β-sheet antiparallel (1670–1685 cm^−1^), and the peak maximum for β-sheet (1615–1635 cm^−1^) was detected, using the function *peakdet* available in MATLAB (Version 2019b). The area was then calculated at the interval of the min/max peak ± 2 cm^−1^.

#### Solubility line (SL)

For determining the SLs, the supernatant of the phase diagrams was measured. For this purpose, 3 µL of the supernatant of each condition was carefully (no air bubbles, no visible aggregates) pipetted on the NanoDrop™ 2000c, and the concentration measured in triplicate. Afterwards, the solubility lines were calculated using a method published by Galm et al*.* [35]. For this study, the conditions with crystals were taken into account. Furthermore, the curves were integrated, using the function integral available in MATLAB (Version 2019a), and the areas from 0 M to 2.5 M salt and from 0 mg/mL to 25 mg/mL protein were calculated.

## Results

This aim of the study was to evaluate the following: (a) whether the influence of different excipients on the long-term protein stability of FT stressed formulations can be followed by the creation of MPPDs and (b) whether the induced instabilities are reversible by a simple heat treatment.

The model system used to perform this investigation consisted of chicken egg white lysozyme, different amounts of NaCl and cryoprotectant. The protein concentrations ranged from 2.5 to 25 mg/mL at pH 5. The NaCl concentration as precipitant was increased up to 2.5 M. Four different cryoprotectants (a) 300 mM sucrose, (b) 1000 mM glycerol, (c) 6.81 mM PEG200, and (d) 0.03 mM Tween20 in a solution containing NaCl were added to this model system separately. Different FT cycle numbers (FT cx; *x* = 0, 1, 3), as well as a heat cycle (FT cy h; *y* = 1, 3), were performed for each of the formulations during the study.

### Multidimensional protein phase diagram (MPPD)

**Fig. 1:**
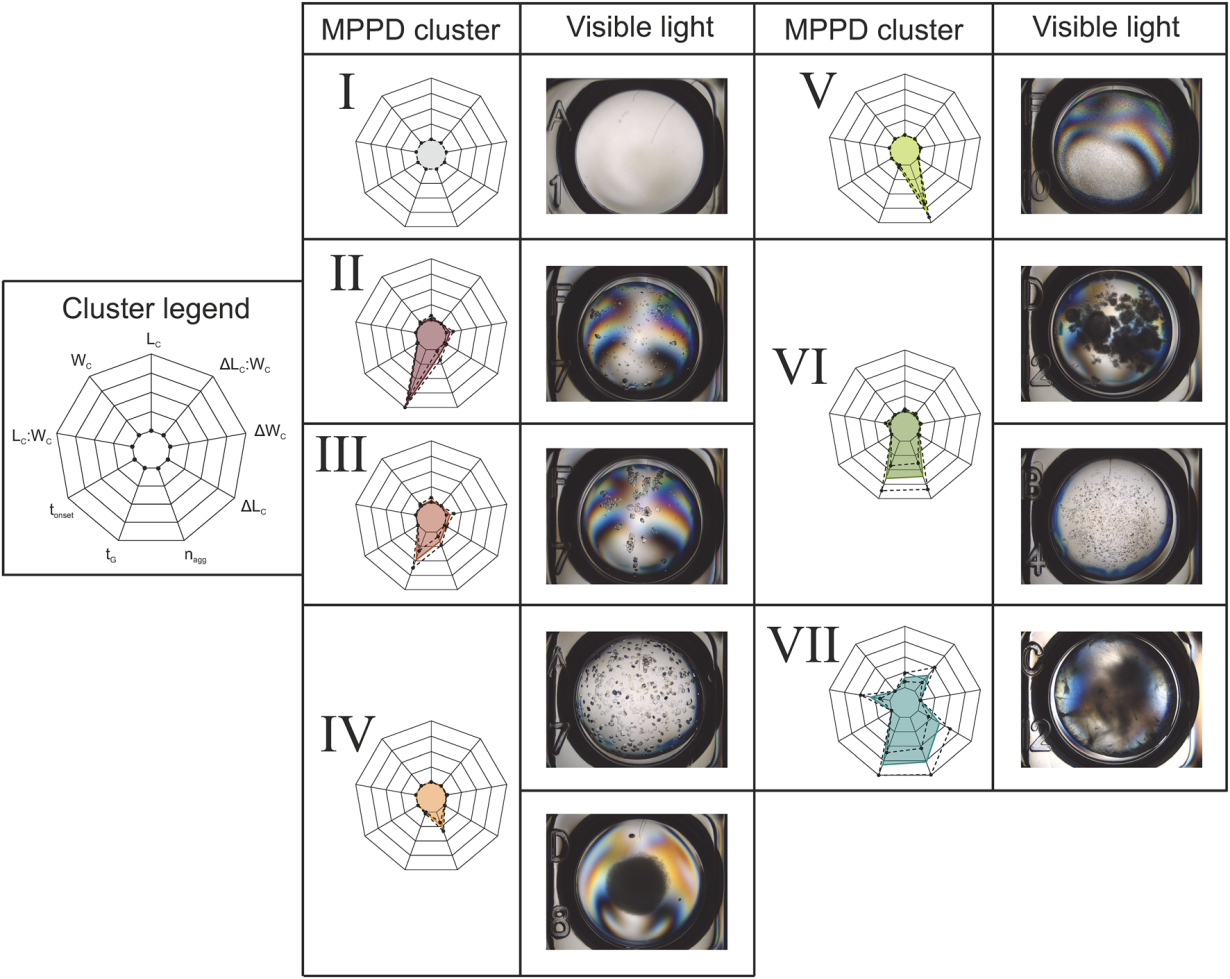
The clusters detected for the data set are shown with the respective example pictures of exemplary crystals. The color surface in the radar charts represents the normalized median values of each image feature. The following image–based features were used for the clusters: the crystal length (*L*_*C*_) and widths (*W*_*C*_), the IQR of the crystal length (*ΔL*_*C*_) and width (*ΔW*_*C*_), the aggregation abundance (*n*_Agg_), the aggregation onset time (*t*_onset_), and aggregation growth time (*t*_G_), as well as the ratio of crystal length and width (*L*_*C*_:*W*_*C*_), and the IQR of this ratio [Δ (*L*_*C*_:*W*_*C*_)]. The median absolute deviation within each cluster for each image–based feature is shown by a dashed line in the radar charts. The absolute cluster values can be found in Table 1. The exemplary crystal pictures are made with visible light. Soluble conditions were presented by Cl I, tetragonal crystals are presented by Cl II, complex structured crystals by Cl III, either tetragonal crystals at FT c0 (top) or dense grown micro crystals with FT stress (bottom) by Cl IV, micro crystals by Cl V, sea urchin (top) and small tetragonal crystal (bottom) by cluster VI, and sea urchin crystals by Cl VII

**Fig. 2:**
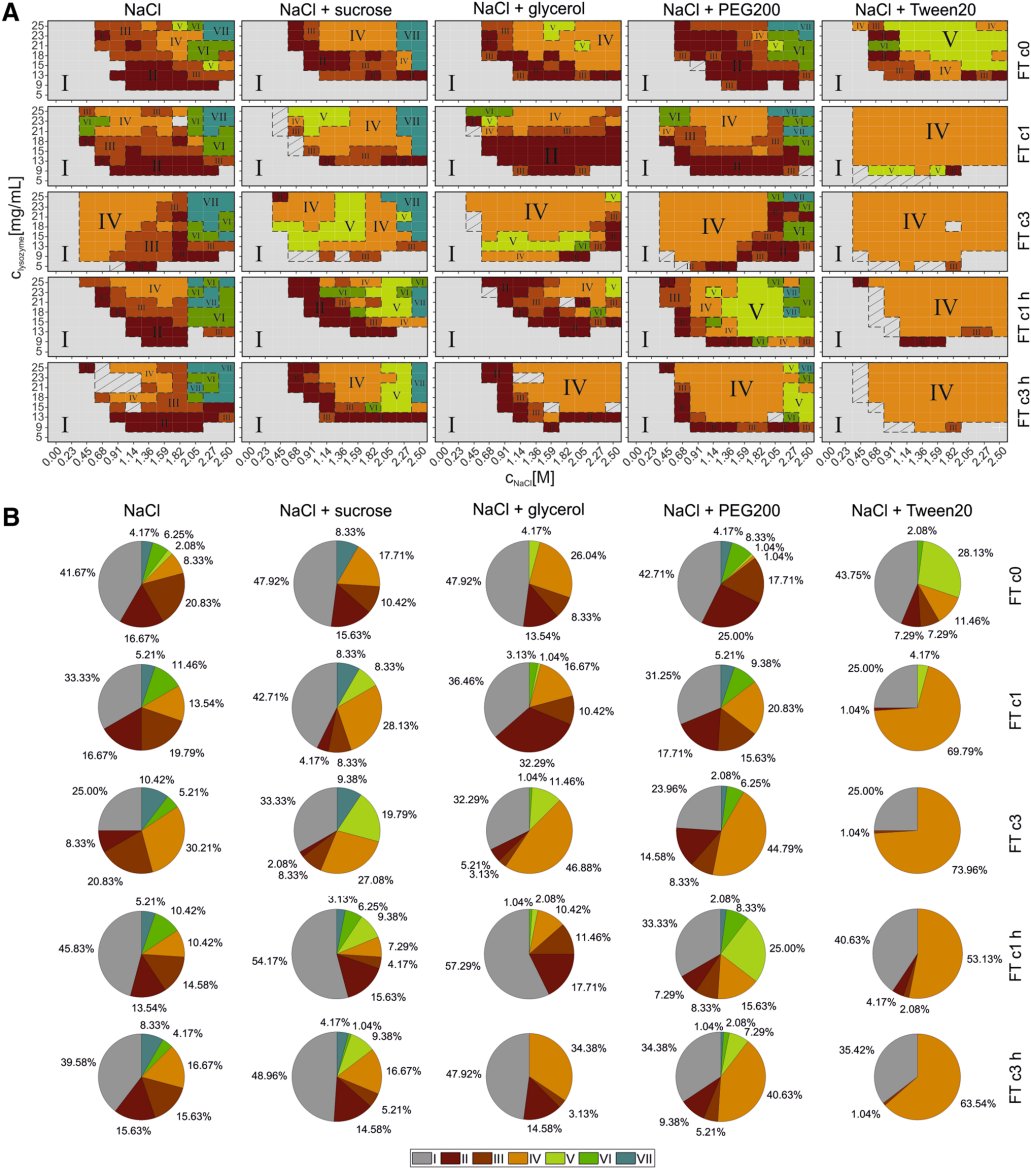
In A the MPPDs for five different lysozyme formulations (NaCl, NaCl + sucrose, NaCl + glycerol, NaCl + PEG200, NaCl + Tween20 (C 1–5)) and different stress protocols (R 1–5). The lysozyme concentration [mg/mL] was varied over the NaCl concentration [M]. Seven clusters were identified and used for the MPPD using the mean cluster color and cluster number similar to the radar charts, shown in Fig. 1. In B the calculated occurrence in [%] of each cluster per phase diagram is shown with the respective values

Overall, 2400 different formulations were studied and resulted in different phase states, soluble and crystalline (exemplary pictures see Fig. 1). To visualize all morphologies and kinetic data in one figure, the MPPD construction includes a data reduction step. This reduction step results in an energy value of 95.5%, which indicates an information loss of 4.5%. An optimal number of seven clusters was obtained when the reduced dataset was clustered; they are shown as radar charts (I-VII) in Fig. 2a. Each radar chart represents a specific combination of image-based features: the crystal length (*L*_*C*_) and widths (*W*_*C*_), variation in crystal length (∆*L*_*C*_) and width (∆*W*_*C*_), the aggregation amount (*n*_agg_), the aggregation onset time (*t*_onset_), and aggregation growth time (*t*_G_), as well as the ratio of crystal length and width (*L*_*C*_: *W*_*C*_), and the variation of this ratio [∆(*L*_*C*_: *W*_*C*_)]. The normalized median values are visualized as a colored surface, and the corresponding median absolute deviation (MAD) is shown as a dashed line in Fig. 1. An overview of the absolute values, representing the median ± MAD, which is calculated based on all formulations within the mentioned cluster, and their ranges of each cluster is given in Table 1.

**Table 1:** Overview of median ± median absolute deviation (MAD) image-based feature values. The values are listed per cluster identified in the separate multidimensional protein phase diagram

	I	II	III	IV	V	VI	VII
*L*_*C*_ [µm]	0 ± 0	202 ± 186	192 ± 209	32 ± 14	34 ± 11	105 ± 92	915 ± 380
*W*_*C*_ [µm]	0 ± 0	141 ± 124	139 ± 150	24 ± 10	25 ± 8	16 ± 9	13 ± 4
*L*_*C*_:*W*_*C*_ [–]	0 ± 0	1.4 ± 0.2	1.3 ± 0.2	1.3 ± 0.1	1.4 ± 0.1	7.8 ± 9.3	76.4 ± 37.9
*t*_onset_ [hours]	0 ± 0	6 ± 9	1 ± 1	0 ± 0	0 ± 0	0 ± 0	0 ± 0
*t*_G_ [hours]	0 ± 0	892 ± 95	456 ± 142	12 ± 15	16 ± 19	620 ± 208	783 ± 195
*n*_agg_ [%]	0 ± 0	10 ± 7	15 ± 7	25 ± 7	77 ± 11	60 ± 22	75 ± 22
∆*L*_*C*_ [µm]	0 ± 0	51 ± 52	52 ± 56	11 ± 7	11 ± 6	37 ± 35	1032 ± 529
∆*W*_*C*_ [µm]	0 ± 0	46 ± 49	48 ± 58	9 ± 6	8 ± 4	7 ± 5	6 ± 4
∆(*L*_*C*_:*W*_*C*_) [−]	0 ± 0	0.3 ± 0.3	0.4 ± 0.3	0.3 ± 0.2	0.4 ± 0.2	3.3 ± 4.2	93.2 ± 43.4

Cluster I (Cl I) represents soluble conditions, all values of the image features are equal to zero, as no aggregation took place. However, for some conditions, which were clustered to Cl I, aggregates were observed. These conditions were analyzed manually and are bordered by dashed lines in Fig. 2. Cl II represents a few (*n*_agg_ = 10 ± 7%) relatively large (*L*_*C*_ = 202 ± 186 µm and *W*_*C*_ = 141 ± 124 µm) crystals which have an onset time after a few hours (*t*_onset_ = 6 ± 9 h) but a very long growth time (*t*_G_ = 892 ± 95 h). An exemplary picture of such a crystal is shown in Fig. 1. Cl III represents crystals which show a slightly higher amount (*n*_agg_ = 15 ± 7%) and an earlier crystal growth onset time (*t*_onset_ = 1 ± 1 h) as well as a shorter growth time (*t*_G_ = 456 ± 142 h). Within Cl IV and Cl V the amount of crystals increased to *n*_agg_ = 25 ± 7% / 77 ± 11%, and the crystal size decreased significantly to *L*_*C*_ = 32 ± 14 µm / 34 ± 11 µm and *W*_*C*_ = 24 ± 10 µm / 25 215 ± 8 µm, receptively (see Table 1 and Fig. 1). Whereas Cl IV indicated two different morphologies: tetragonal crystals and densely grown micro crystals (see Fig. 1). The tetragonal crystals grew preferably when no stress was applied, whereas the micro crystals grew after FT stress was applied. Cl VI showed a significant higher crystal growth time (*t*_G_ = 620 ± 208 h) and a significant increased ratio between crystal width and length (*L*_*C*_:*W*_*C*_ = 7.8 ± 9.3). This cluster also represented two types of crystal morphologies: sea urchin crystals and small tetragonal crystals. The sea urchins are dominant in the supersaturated region, and the small tetragonal crystals grow at lower salt and protein concentrations. Cl VII showed the highest values for the crystal length and the ratio between crystal length and width, as well as crystal growth time (*L*_*C*_ = 915 ± 380 µm, *L*_*C*_:*W*_*C*_ = 76.4 ± 37.9, *t*_G_ = 783 ± 195 h).

### Formulations

The results of the long-term storage experiment are presented in the MPPD in Fig. 2, where lysozyme was monitored for 40 days at 20 °C using 2400 different formulations. The different columns (C 1–5) in Fig. 2a represent the different formulations with NaCl (C 1), NaCl + 300 mM sucrose (C 2), NaCl + 1000 mM glycerol (C 3), NaCl + 6.81 mM PEG200 (C 4), and NaCl + 0.03 mM Tween20 (C 5), whereas the different rows (R 1–5) represent the different cycles performed [FT c0, FT c1, and FT c3 (R 1–3)] and the combination of FT stress followed by a heat cycle [FT c1 h and FT c3 h (R 4–5)]. Figure 2b summarizes the percentages occupied by the different clusters for each system.

#### Initial state–FT c0

##### NaCl

All clusters were present at FT c0, whereas Cl II and Cl III were dominant in the transition zone from the soluble (Cl I) to the aggregation zone, see Fig. 2a, R1. Increasing the NaCl and lysozyme concentration resulted in Cl IV, Cl V, and Cl VI. At the highest lysozyme concentrations (23 mg/mL and 25 mg/mL) and NaCl concentrations (2.27 M and 2.5 M), Cl VII appeared. The overall contribution of the different clusters were for Cl I 47.92%, Cl II 15.63%, Cl III 20.83%, Cl IV 8.33%, Cl V 2.08%, Cl VI 6.25%, and Cl VII 4.17%, see Fig. 2b, R 1 and C 1.

##### Sucrose

Adding sucrose to the formulations, the aggregation zone slightly decreases for FT c0, and Cl V and VI disappeared, see Fig. 2a, C 2. Cl IV was present at lysozyme concentrations above 18 mg/mL and NaCl concentrations between 1.14 M and 2.05 M. The transition zone from the soluble zone (Cl I) to the aggregation zone consisted of Cl II and Cl III. Cl VII was present at the highest lysozyme and NaCl concentrations. The overall contribution of the different clusters was for Cl I 41.67%, Cl II 16.67%, Cl III 20.83%, Cl IV 8.33%, Cl V 2.08%, Cl VI 6.25%, and Cl VII 4.17%, see Fig. 2b, R 1 and C 2.

##### Glycerol

Replacing sucrose by glycerol in the formulation resulted in a smaller aggregation zone/larger soluble zone (Cl I) compared to pure NaCl formulations for FT c0. In the transition zone to Cl I was still created out of Cl II and Cl III, whereas it was smaller compared to NaCl formulations. In the remaining aggregation zone, Cl IV was dominant, see Fig. 2a, C 3. The overall contribution of the different clusters were for Cl I 47.92%, Cl II 13.54%, Cl III 8.33%, Cl IV 26.04%, and Cl V 4.17%, see Fig. 2b, R 1 and C 3.

##### PEG200

PEG200 formulations showed phase behaviors very similar to those of NaCl formulations, see Fig. 2a, C 4. In the transition zone from the soluble zone (Cl I) to the aggregation zone, Cl II, and at increasing protein and salt concentrations, Cl III was dominant up to a NaCl concentration of 1.82 M. In the high concentration region, mainly Cl VI and VII were created. The overall contribution of the different clusters were for Cl I 42.71%, Cl II 25.00%, Cl III 17.71%, Cl IV 1.04%, Cl V 1.04%, Cl VI 8.33%, and Cl VII 4.17%, see Fig. 2b, R 1 and C 4.

##### Tween20

For Tween20 formulations Cl V was most dominant at FT c0, see Fig. 2a, C 5. The occurrence of Cl I was slightly lower compared to that of the NaCl formulations. The overall contribution of the different clusters were for Cl I 43.75%, Cl II 7.29%, Cl III 7.29%, Cl IV 11.46%, Cl V 28.13%, and %, Cl VI 2.08%, see Fig. 2b, R 1 and C 5.

#### FT cycles–FT c1and FT c3

In general increasing the number of FT cycles resulted in a decrease in the Cl I region (soluble region) for all formulations tested; see Fig. 2b, R 1–3. The more FT cycles were applied, the higher the amount of Cl IV was observed.

##### NaCl

NaCl formulations showed an increase of condition belonging to Cl IV when increasing the cycle number, 8.33% to 13.54% to 30.21%, see Fig. 2b, C 1. Cl IV at FT c1 was created at lysozyme concentrations higher than 21 mg/mL up to 1.59 M NaCl. At FT c3 the region expanded and Cl IV was dominant at all lysozyme concentrations up to 1.59 M NaCl, see Fig. 2a, R 3. Considering that, the higher the lysozyme concentration, the more salt had to be added to create Cl IV. At high supersaturations, still Cl VI and Cl VII were dominant for FT c1 and FT 3.

##### Sucrose

Sucrose formulations do also show a cluster transformation to Cl IV and Cl V by increasing the cycle number, see Fig. 2a, C 2. The occurrence of these clusters increased from 17.71% to 28.13% to 27.08% for Cl IV and from 0% to 8.33% to 19.97% for Cl V while increasing the number of cycles, see Fig. 2b, R 1–3 C 2. Regarding the positions of the clusters, the Cl IV region is spilt by Cl V, see Fig. 2a, C 2. The occurrence of Cl VII stayed the same (8.5%), whereas the region with Cl II and Cl III decreased significantly. For Cl II the occurrence decreased from 15.63% to 4.17% to 2.08% and for Cl II from 10.42 to 8.33% for FT c1 and FT c3, see Fig. 2b, C 2. The Cl I zone, however, was not significantly decreased at FT c1, compared to NaCl formulations, only slightly decreased at FT c3 (47.92% to 42.71% to 33.33%).

##### Glycerol

Formulations containing glycerol showed a significant increase of Cl II from FT c0 to FT c1, 13.54–32.29%, respectively, see Fig. 2a, C 3 and Fig. 2b, C 3. At FT c3 a cluster transformation to mainly Cl IV (46.88%) took place. The Cl I region was slightly decreased when the number of cycles was increased (47.92% to 36.46% to 32.29%).

##### PEG200

PEG200 formulations showed the same cluster transition as NaCl formulations, see Fig. 2a, C 4. Concerning the occurrence of the clusters small differences were seen. When the formulations were stressed, the occurrence of Cl IV was dominant, whereas Cl II and Cl III were less present compared to NaCl formulations. At FT c3 44.79% of the conditions belong to Cl IV, whereas only 30.21% belong to this cluster when only NaCl was in the formulation, see Fig. 2a, C 4 and b, C 4.

##### Tween20

Tween20 formulation did show a cluster transformation to Cl IV from FT c0 with an occurrence of 11.46% to FT c1 with an occurrence of 69.79%. To FT c3 the occurrence of Cl IV slightly increased to maximum of 73.96%, see Fig. 2a, C 5 and Fig. 2b, C 5. The occurrence of the soluble zone (Cl I) decreased significantly from 43.75% at FT c0 to 25.00% at FT c1 and FT c3.

#### Heat cycle–FT c1 h and FT c3 h

The reversibility regarding the phase state (soluble/aggregate) and the occurrence of Cl II and Cl III were analyzed. In general, the additional heat cycle increases the Cl I region compared to the corresponding FT cycles, see Fig. 2a R 4–5.

##### NaCl

For NaCl formulations similar positions of the cluster at FT c1 h and FT c3 h compared to FT c0 could be observed. Regarding the reversibility of the phase states, the occurrence of Cl I reached values similar to those at FT c0 (41.67%) at FT c1 h with 45.83% and at FT c3 h with 39.58%, see Fig. 2a, C 1 and Fig. 2b, C 1. The occurrence of Cl II and Cl III did not significantly change between all the cycles applied. The Cl II and Cl III appeared again adjacent to Cl I as it was the case for FT c0, when a heat cycle was performed.

##### Sucrose

When sucrose was added to the formulations and heat cycling was performed after the formulations were FT–stressed, a significant zone of Cl II was created in zone adjacent to Cl I which was not the case at FT c1 and FT c3, see Fig. 2a, C 2. The occurrence of these clusters was very low at FT c1 and FT c3, but Cl II reached a similar occurrence compared to FT c0 (16.67%), when a heat cycle was performed (FT c1 h 13.54%, FT c3 h 15.63%), see Fig. 2b, R 4–5 and C 2. The occurrence of Cl I increased significantly for FT c1 h and FT c3 h (45.83% and 39.58%) compared to the respective systems at FT c1 and FT c3 (33.33% and 25.00%), whereas nearly the same occurrence as at FT c0 (41.67%) with sucrose were reached, see Fig. 2a, C 2 and Fig. 2b, C 2.

##### Glycerol

Applying a heat cycle to glycerol formulations after they were stressed with freezing/thawing resulted in an appearance of the same clusters as occurred at FT c0, as well as the position of these clusters are similar, see Fig. 2, C 3. Concerning the occurrence of Cl I, the same occurrence as at FT c0 (47.92%) could be reached at FT c3 h (47.92%) and was even increased at FT c1 h (54%). In addition the occurrence of Cl II and Cl III (FT c1 h 17.71%/11.46%, FT c3 h 14.58%/3.13%) reached values close to those at FT c0 (Cl II 13.54%, Cl III 8.33%), with the exception of Cl III at FT c3 h (3.31%), see Fig. 2b R 4–5 and C 3.

##### PEG200

The addition of a heat cycle to PEG200 formulations resulted in a similar positioning of the Cl II and Cl III region compared to FT c0, which was adjacent to Cl I. Nevertheless, this region was significant at FT c1 h (Cl II 7.29%, Cl III 8.33%) and FT c3 h (Cl II 9.38%, Cl III 5.21%) smaller than at FT c0 (Cl II 25.00%, Cl III 17.71%), see Fig. 2a, C 4 and Fig. 2b. The occurrence of Cl I for FT c1 h (33.33%) and FT c3 h (34.38%) only reached values of FT c1 (31.25%) and not the one of FT c0 (42.71%).

##### Tween20

The heat cycle influenced only the occurrence of Cl I for Tween20 formulations and not the cluster formation itself. Still mainly Cl IV was observed like at FT c1 and FT c3. At FT c1 h a similar occurrence of 40.63% of Cl I compared to FT c0 with 43.75% could be observed. FT c3 h showed a slightly smaller occurrence of Cl I with 35.42% compared to FT c0, see Fig. 2a, C 5 and Fig. 2b, C 5.

#### Formulation comparison

Adding different excipients to a NaCl phase diagram resulted in a different phase behavior for each excipient, see Fig. 2a, R 1/C 1–5. In the following, a comparison with a focus of the effects arising from the different excipients is laid for the initial state and the different cycles studied. In general, the influence of FT cycles and the reversibility by heat is strongly dependent on the cryoprotectant.

##### Initial state–FT c0

In the transition zone to Cl I for all formulations, Cl II and Cl III are present, whereas for Tween20 formulations, the region is very small. Regarding the size of the Cl I region, NaCl and PEG200 formulations showed a slightly smaller region than formulations containing sucrose, glycerol or Tween20, see Fig. 2b, R 1. Cl VII was only created in the highly concentrated region for NaCl, sucrose and PEG200 formulations.

##### FT cycle–FT c1 and FT c3

The influence of the FT cycles, as well as the phase behavior at FT c0, were very similar for NaCl and PEG200 formulations. The position of the clusters changed similar when FT stress was applied, see Fig. 2a, R 1–3, C 1 and C 4. The occurrence of Cl I was highest with sucrose and glycerol formulations compared to the other formulations tested, see Fig. 2b. Furthermore, the FT stress did not have such a significant impact on the occurrence of Cl II for sucrose and glycerol formulations. In general, the higher the cycle number, the lower the occurrence of Cl I. For Tween20 formulations there was no difference between FT c1 and FT c3 regarding the Cl I occurrence visible, see Fig. 2b, R 1–3.

##### Heat cycle–FT c1 h and FT c3 h

When a heat cycle was performed the increase of the Cl I region when a heat cycle was added was less pronounced for PEG200 and Tween20 formulations compared to the other formulations tested. All formulations showed cluster transformation back to the clusters seen at FT c0, with the exception of PEG200 and Tween20 formulations, see Fig. 2a, R 4–5 and Fig. 2b, R 4–5. With a heat cycle, Cl V occurred for PEG200 formulations, which was not seen at any other system with PEG200 formulations. With Tween20 the heat cycle only influenced the occurrence of Cl I and did not result in a cluster transformation at all.

### Analytics

Information of three different analytic methods: (1) protein solubility (SL), (2) protein size (DLS) and (3) protein structure (FTIR) were added to the MPPDs. These measurements were performed for two reasons: (a) to evaluate if initial stress leads to changes in structural parameter or size of the proteins under investigation and (b) to evaluate if the process exploiting heat reversibility leads of structural (FTIR) or size (DLS) based alterations.

#### Solubility line (SL)

**Fig. 3:**
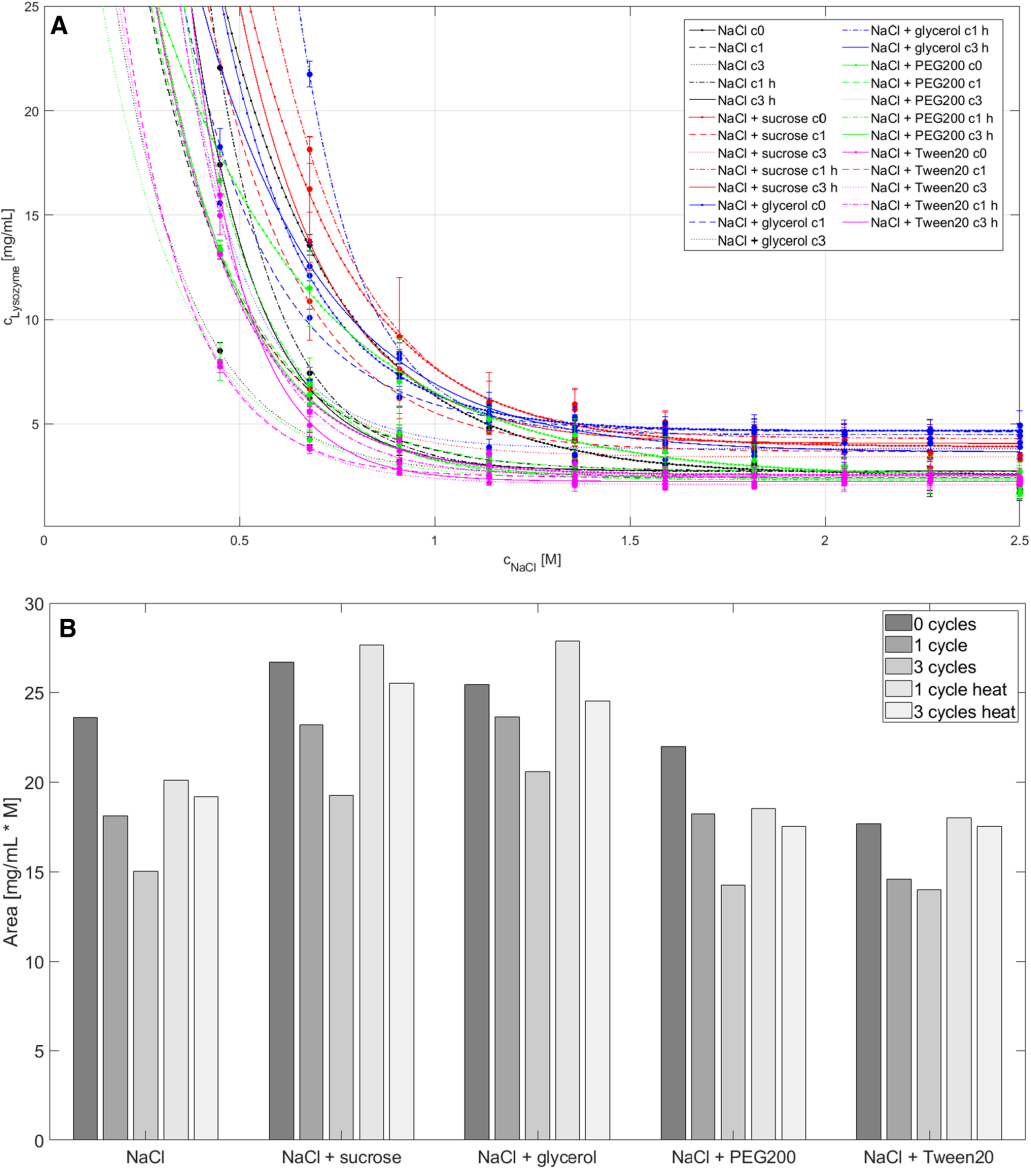
In A the solubility lines of lysozyme in different formulations where different stress protocols were applied. The lysozyme concentration in mg/mL is plotted over a varying NaCl concentration in M. In B the calculated area of each phase diagram investigated is shown. The SL area is plotted over the different formulations tested, the different shades of gray represents FT c0 (darkest gray), FT c1, FT c3, FT c1 h, FT c3 h (lightest gray) (color figure online)

To investigate the protein solubility, supernatant measurements of each phase diagram were performed and SLs were calculated, as described in “Solubility line (SL)” and presented in Fig. 3a. To identify more easily differences of the SLs, the area was calculated underneath the curves as described in “Solubility line (SL)”. Bar graphs of these values are shown in Fig. 3b. The trend regarding the influence of FT stress and the heat reversibility are similar for all lysozyme formulations tested. For the formulations with NaCl and the formulations where PEG200 was added, the area was the largest without any FT stress, see Fig. 3b. The smaller the calculated SL area, the more the SL is shifted to lower protein and salt concentrations. A large SL area indicates high protein solubility and a small aggregation zone in the respective phase diagram. In formulations where sucrose or Tween20 were added, the area of FT c0 was similar to that of FT c1 h. With glycerol, the area with the formulations which performed FT c1 h was even larger than for FT c0. An overall trend could be observed when the influence of FT cycles was taken into account. The more the FT cycles were applied to the formulations, the smaller the SL area became. In addition, the heat cycle results in a significant increase in the SL area for FT c1 and FT c3. The only exception was shown with Tween20 formulations. Here, the heat cycle did not increase the SL area for FT c1, see Fig. 3b.

Comparing the different excipients to each other, the formulations with sucrose addition showed the highest SL area values, followed by glycerol formulations, see Fig. 3b. These formulations are followed by NaCl, NaCl + PEG200 and NaCl + Tween20 formulations in the order from high to low SL area values.

#### Dynamic light scattering (DLS)

**Fig. 4:**
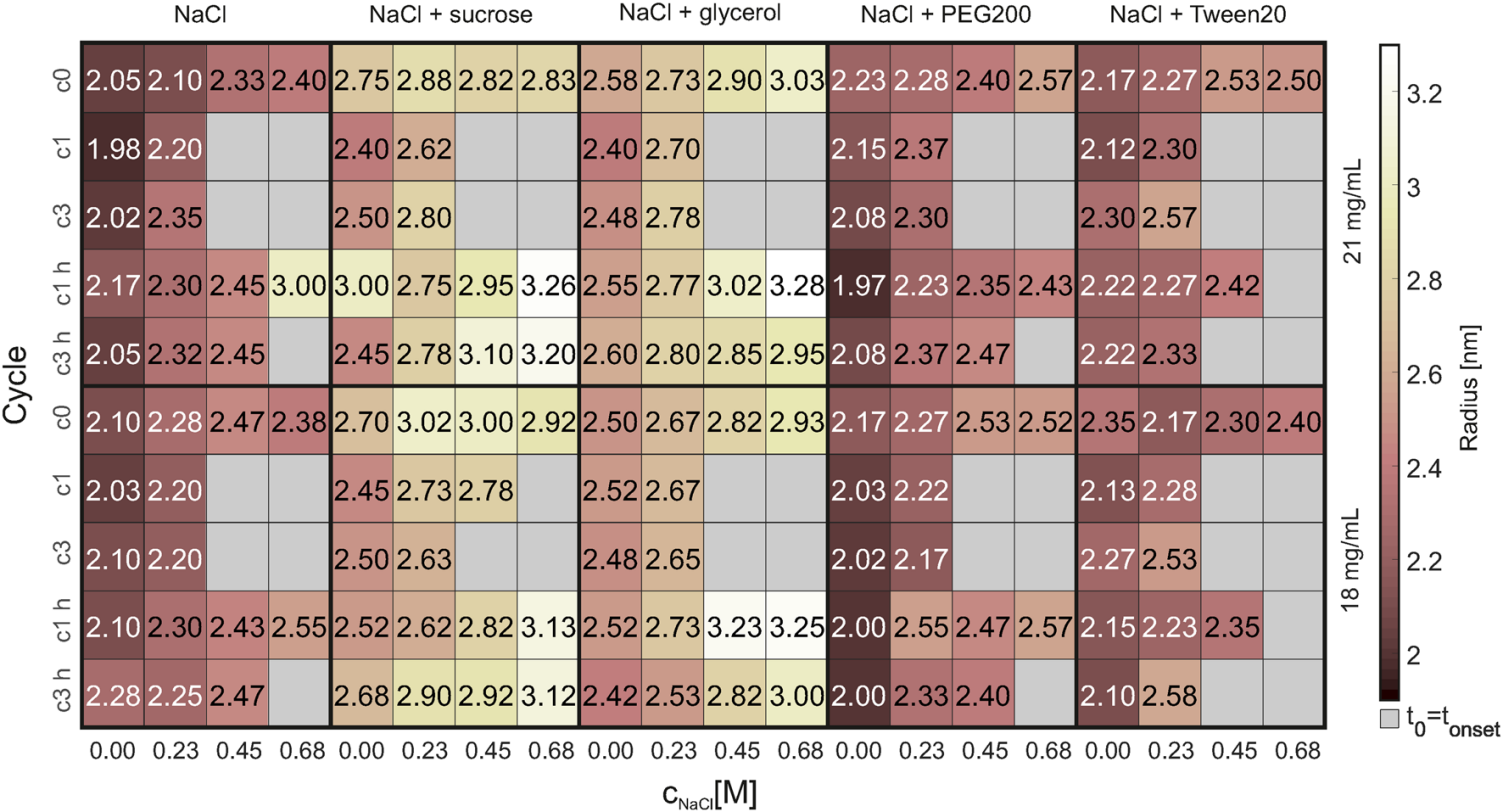
The results of the 151 DLS measurements are shown. The NaCl concentration is plotted over the cycle number for the different formulations investigated. The upper row shows the results for 21 mg/mL lysozyme and the lower row for 18 mg/mL. The color bar represents the apparent hydrodynamic radius in nm. Measurements of conditions where the box is marked gray were not possible due to aggregation appearance. The respective deviations from the triplicate measurements are shown in the “Supplementary Material” Figure S2 (color figure online)

The protein size of the formulations investigated was measured performing DLS measurements in triplicate, see Fig. 4. No significant changes could be observed. The stress type (FT and the heat) and amount (cycle number) did not influences the protein size. Comparing the results of the different formulations to each other, small differences were observed: Formulations with NaCl, PEG200, and Tween20 show radii in a lower range (app. 2.0–2.5 nm), whereas formulations with sucrose or glycerol show radii with approximately 2.3–3.3 nm. Finally, heat reversibility did not show significant alteration in the size of the protein when compared to the initial dimensions.

#### Fourier-transform infrared spectroscopy (FTIR)

**Fig. 5:**
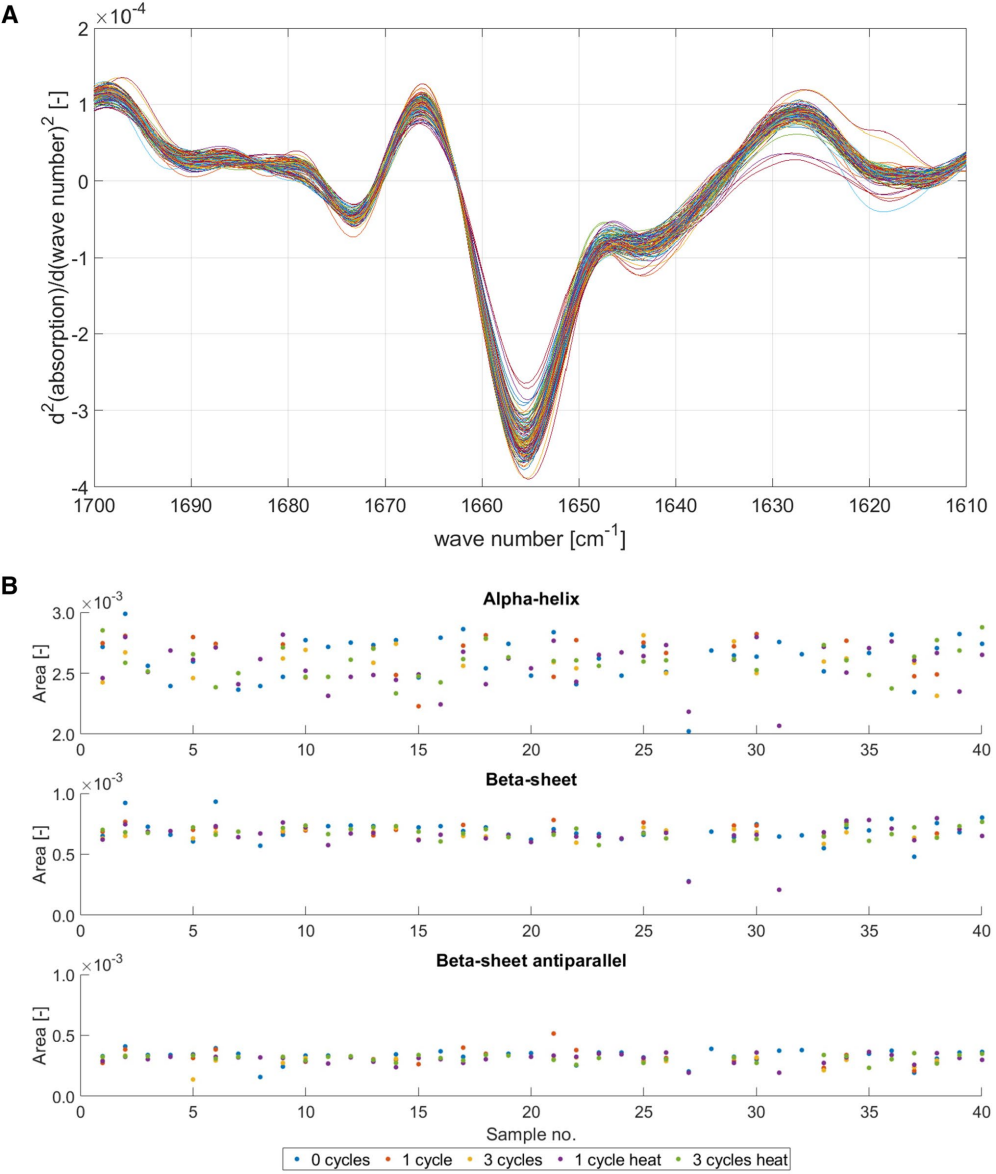
In A the pre–processed FTIR spectra for all 151 samples, which performed also the DLS measurements, are plotted (see Fig. 4). The range between a wave number of 1650 and 1685 cm^−1^ represents alpha–helix structure, 1615 and 635 cm^−1^ beta–sheet structures, and 1670 and1685 cm^−1^ beta–sheet antiparallel structures. In B the calculated areas of the interesting regions for alpha–helix (top), beta–sheet (middle) and beta–sheet antiparallel (bottom) are shown for all measured samples (Sample no. 1–8: NaCl; 9–16: NaCl + sucrose; 17–24: NaCl + glycerol; 25–32: NaCl + PEG200; 33:40: NaCl + Tween20). Blue dots represent 0 cycles, red points 1 cycle, yellow dots 3 cycles, purple dots 1 cycle heat, and green dots 3 cycle heat (color figure online)

Protein structure information were investigated using FTIR. All samples measured with DLS were also analyzed using FTIR. After data pre-processing (see “Fourier-transform infrared spectroscopy (FTIR)”), the spectra is shown in Fig. 5 A. In the interesting amid I region where the alpha–helix (1650–1685 cm^−1^), beta–sheet (1615–1635 cm^−1^) and beta–sheet antiparallel (1670–1685 cm^−1^) structures absorb nearly no differences were detected. This becomes clearer when the calculated areas for each region are compared to each other, see Fig. 5b. With these results, no influence of protein and salt concentration, stress type (FT or heat cycling), FT cycle number, and excipients could be determined in this study.

## Discussion

In the following section, results are discussed concerning the influence of the tested excipients (300 mM sucrose, 1000 mM glycerol, 6.81 mM PEG200, 0.03 mM Tween20) on the long–term protein stability of FT–stressed formulations, as well as whether these changes are reversible by adding a heat cycle after the respective FT protocol was performed. The results are discussed separately for each formulations tested and the respective influence of the heat cycle. Finally, a comparison is made across the different formulations.

### Multidimensional protein phase diagram (MPPD)

The creation of the MPPDs resulted in an information loss of only 10% due to data dimension reduction from nine to three dimensions. Consulting literature, this falls in an acceptable range [36].

The MPPD procedure allowed an automated evaluation and clustering providing a rapid overview over a huge data set of complex phase transitions taking place in the phase diagrams. As shown in Fig. 2 it further provided insight into positioning (Fig. 2a) and occurrence (Fig. 2b) of different phase states. While clustering as such occurs in an automated fashion, the choice of suitable descriptors is of course subjective and great care needs to be taken when deciding on a certain set. Two examples underlining this are seen in the current study. The misclassifications seen for Cl I (meant to represent soluble formulations only) were due to accommodating the high variety of crystal sizes in a single data set. The data set included length and width values, which are very large (due to sea urchin crystals) and very small (due to micro crystals).

With the normalizing step during MPPD creation, the small size values close to zero let to an incorporation into Cl I. In addition, the formulations belonging to a cluster do show a distinct distribution and the transition from one to another cluster might lead to overlaps. This is visualized in Figure S1 in the Supplementary Material, where all 2400 conditions are plotted.

A second issue going hand–in–hand with the above findings is the appearance of two different morphologies seen for Cl IV and Cl VI, as shown in Fig. 1. Here, the reason lies clearly in the similarity of the image-based parameters taken into account, namely size and time. This shows the care and orthogonality needed when choosing descriptors for an automated classification scheme. In the present study, a distinction of different morphologies could be possibly reached by an addition of the crystal intensity, due to the higher intensity of sea urchin crystals and the dense micro crystal when compared to tetragonal crystals (independent of size).

Nevertheless, a MPPD creation allows an objective scoring and clustering of phase diagrams, based on crystal dimensions and other descriptors chosen. In a second step, however, the raw data of the created clusters needs to be checked to ensure that choice of descriptors, overlap, and distribution of features does not lead to a false interpretation of data.

### Formulations

In the following section, the results obtained for each system tested using the MPPD approach are discussed. The evaluation of molecular starting conditions, namely the DLS and FTIR measurements of the soluble regions chosen (Figs. 4, 5) for two different concentrations show no significant alteration in measured values as a function of stress applied. From this, it can be concluded that the formation of different crystal morphologies and thus cluster distribution is not dependent on structural protein parameter. But it should keep in mind that a small fraction of protein might undergo partial unfolding and/or aggregation, which is not detectable using the described DLS or FTIR measurements. These small changes might cause undesirable particle formation and change in the crystallization kinetics.

The increase in apparent size seen for increasing salt concentrations, and respectively higher ionic strengths, within a certain formulation subgroup might be due to increasing hydrophobic protein–protein interactions [37]. The greater the protein–protein interactions, the lower the molecule diffusion in the solutions and the bigger the hydrodynamic radius estimated by DLS measurements [38]. The slightly higher apparent radii of sucrose and glycerol formulations can be explained by an overall increased viscosity, due to the high excipient concentrations used, and the procedure of how the hydrodynamic radius is estimated (Stokes–Einstein equation) [38, 39].

The respective excipients were chosen due to their different protein interaction mechanism, which are explained in the following.

#### Salt

Using NaCl as an additive only, the salt concentration mainly modulates electrostatic interactions and suppressing these allows hydrophobic interactions to play a more dominant role. Lysozyme with a pI of 11.35 [40] is positively charged at the operating pH of pH 5. Given this, the distance to its pI seems wide enough so that a slight change in pH will not result in changes of surface charge and effects seen can be related to alteration in additive solely. The concentration range from 0 to 1.1 M NaCl was chosen due to the ability of stabilizing or destabilizing the protein stability depending on the protein surface charge and the salt concentration [41].

#### Osmolytes

Two different osmolytes were chosen in this study, due to their ability to stabilize the native structure of the protein upon environmental stress [42–44] and thus also act as cryoprotectants. The osmolytes used in this study are sucrose [45, 46], a sugar, and glycerol [43, 47], a polyol.

##### Sucrose

Sucrose is known to be an effective cryoprotectant, due to stabilizing the native structure of proteins by thermodynamic stabilization. Thereby, preferential exclusion of sucrose and subsequently hydration of the protein surface are taking place [48].

##### Glycerol

The mechanisms triggered by adding glycerol are not completely understood. The most significant contributions are twofold. There is preferential exclusion effect of glycerol, comparable with sucrose, where the native protein structure is stabilized. Furthermore, stabilization of glycerol is assumed to be also due to preferential interaction of glycerol and the hydrophobic regions on the protein surface and following the inhibition of protein unfolding [47].

##### Polymers

Polymers are also known to generally stabilize protein solutions [30, 49], whereas the mode of action is strongly dependent on their molecular weight [50] and concentration applied [51]. Low molecular weight PEG present in low concentrations may induce protein stabilization due to the steric shielding of attractive protein–protein interactions [44, 51]. This effect is exploited in this study using 6.81 mM (6 w/w%) PEG200. Depending on the hydrophobicity of the protein surface, the mechanism of PEG is influenced. When the protein surface is hydrophobic, destabilizing preferential interaction of the hydrophilic PEG molecules and the hydrophobic patches on the protein surface is taking place. Otherwise, like in this study where the protein surface is positively charged, stabilizing preferential exclusion of the PEG molecules is taken place [49].

##### Surfactant

Finally surfactants, and in this group of excipients especially Tween20 and Tween80 are commonly used, due to their ability to stabilize protein stability against freeze stress–induced aggregation [20, 52]. In this study, 0.03 mM of Tween20 was chosen. The chosen concentration was distinctly below the critical micelle concentration (CMC) of 0.57 mM [53] to potentially prevent surface loss and aggregation [54]. Surfactants are known to interact with the hydrophobic regions on the protein surface [54–56]. In addition, surfactants are also known to prevent the unfolding of the protein on hydrophobic surfaces such as air–water [57].

#### Initial state–FT c0

Using NaCl as an additive, the salt concentration influenced the phase behavior, the aggregation kinetics, the crystal morphology, and the radius of lysozyme. Here we clearly see the interplay of electrostatic and hydrophobic interactions. In short, at lower salt concentrations, long–range repulsive electrostatic protein interactions are significant [33, 58]. These forces are reduced by the presence of salt ions and short–range attractive forces become dominant, which results in aggregation [22, 37, 59, 60]. Consequently, at low NaCl concentrations (< 300 mM), a salting–in (stabilizing) effect and at high NaCl concentrations, a salting–out (destabilizing) effect was observed [41].

The salt concentration not only determined protein solubility but also influenced the crystal morphology. The aggregation zone occurs adjacent to Cl I, representing the soluble zone. This zone is often referred to as the labile or crystallization zone; here, the energy barrier to create nuclei is overcome and crystal growth can occur [8]. As the appearance of Cl II and Cl III corresponded to this zone, they showed similar crystal sizes but the growth time differs.

For Cl III the growth time (*t*_G_) is smaller, however, this cluster was mainly seen for higher lysozyme concentrations, thus a higher supersaturation [61] and as a consequence enhanced creation of critical nuclei [62, 63]. Higher supersaturation is assumed to correlate to shorter growth time, which results of crystal growth to bigger sizes. Bigger crystals were not reached in the phase diagram, here, it is assumed that the concentration steps of lysozyme and NaCl were too huge to reach this zone after Cl II and Cl III appeared. Instead, the supersaturation was too high for supporting crystal growth but with increasing salt concentrations and high protein concentrations, resulted in an increase in the amount of crystals and a decrease in crystal size [61, 64, 65], corresponding to Cl IV and Cl V, see Table 1. At very high lysozyme and NaCl concentrations, the supersaturation reaches a level where the growth of unstable polymorph crystals (sea urchin crystals) is promoted [17, 66]. This morphology is represented partly by Cl VI and mainly by Cl VII.

When adding sucrose representing the group of osmolytes to the salt containing systems at FT c0 a slightly higher lysozyme solubility was reached, whereas the size of the aggregation zone slightly decreased. Subsequently, a small stabilizing effect can be attributed, due to the mentioned preferential exclusion of sucrose [48].

Regarding cluster positioning and occurrence, the transition to the soluble zone seems unaltered, while the aggregation shape changed to smaller crystals (from Cl III to Cl IV) at higher protein and medium NaCl concentrations, when sucrose was added. The supersaturation level is high enough to create critical nuclei but due to preferential exclusion of sucrose, the growth time (*t*_G_) is reduced, which results in a higher amount of small crystals. In addition, at higher NaCl concentrations, the zone of Cl VII is larger adding sucrose to the formulations when compared to pure NaCl formulations. This might be due to the higher viscosity of sucrose formulations in this region compared to pure NaCl formulations. The viscosity of a 300 mM sucrose solution (~ 10 w/w %) at 25 °C is 1.31 mPas which is higher than the viscosity of pure water at 25 °C of 0.89 mPas [67]. Due to the lower nucleation rate, high supersaturation in these formulations and the formation of temporary LLPS, sea urchin crystals grow preferably in this region [17, 66].

Comparable to the addition of sucrose, we see an increase of Cl I (soluble region) when adding glycerol. A higher viscosity of the formulation might also explain the increasing amount of small crystal sizes at intermediate salt concentrations. The viscosity at 25 °C of a 1000 mM glycerol (~ 9 w/w%) solution is slightly lower (1.15 mPas) compared to a 300 mM sucrose formulation (1.31 mPas) [67]. The cluster formation only differed in the high-salt region compared to sucrose formulations. No sea urchins (Cl VI or Cl VII) appeared with glycerol formulations, see Fig. 2a, C 3. Glycerol seems to influence the nucleation rate in this region. Nevertheless, due to the still very high nucleation rate micro crystals grow (Cl IV).

PEG200 representing the group of polymers is known to be preferentially excluded. However, according to the size of the aggregation zone, lysozyme solubility, and cluster formation, no significant changes were observed compared to the pure NaCl formulations. In the applied concentration (as compared to protein and salt concentration present) PEG200 is probably to low concentrated for non-stressed conditions to lead to a significant change. Due to NaCl attractive protein–protein interactions occur, which can be shielded by the PEG molecules. However, in this case it is assumed that the attractive protein–protein interactions are too present and/or the PEG concentration is too low to stabilize lysozyme. A higher PEG concentration or molecule weight is assumed to be more effective [50, 51].

Finally, the influence of surfactants was probed by adding Tween20. The protein phase behavior was not changed significantly by the addition of Tween20, but the morphology and the protein solubility differ compared to pure NaCl formulations. The interaction of Tween20 probably resulted in lower protein solubility, and the SL area showed smaller values, see Fig. 3b, due to the shifted equilibrium between monomer and aggregated proteins towards aggregated proteins. The interaction of Tween20 with the hydrophobic patches on the protein surface [54–56] might have resulted in a significant cluster formation of Cl V, indicating micro crystals. It can be assumed that, due to the interaction of Tween20 on the protein surface, the formation of bigger tetragonal crystals is inhibited. Finally, unfolding on hydrophobic water–air interfaces seems to be not a problem for lysozyme in this study and subsequently, no stabilizing by Tween20 at FT c0 could be observed.

#### FT cycle–FT c1 and FT c3

The unmet ability of the MPPD approach to visualize positioning (Fig. 2a) and occurrence (Fig. 2b) of cluster transformation can also be seen in the development of cluster during FT cycling. In general, all tested excipients had an impact on the MPPD and the SL area compared to the pure NaCl formulations. In the following, the potential mechanistic processes occurring (shielding attractive protein–protein interactions, preferential hydration, and the stabilization of the native state) and thus being the driver behind the cluster transformations during FT cycling are discussed. For all tested excipients, a reduction of Cl I is seen and can be linked to freeze concentration effects experienced during FT cycling. Freeze concentration results in an increase of supersaturation, leading to an increase in the concentration of all solutes, such as buffer components, excipient, and proteins, due to the formation of ice crystals [11, 17]. This results in protein aggregation [12, 13] and subsequently, the protein solubility is lowered due to the shifted aggregate/monomer equilibrium towards aggregates, which was observed with the solubility lines, see Fig. 3b. In addition to the effect of freeze concentration, the decrease in Cl I can also be attributed to the repetition of the FT stress effects as such. In combination, next to freeze concentration [11, 17], temperature-induced LLPS [9, 17, 18] and denaturation on the water–ice interface [7, 20] might take place. Among these effects, however, denaturation on the water–ice interface can be excluded, at least for the measured samples, due to the shown similarity of the FTIR spectra to those of FT c0 (Fig. 5).

A cluster transformation towards a higher portion of Cl IV and shift towards lower salt concentrations was shown for NaCl formulation (and all other systems discussed below), when FT stress was applied, see Fig. 2. This indicates a change to more and smaller crystals, pointing towards a freeze concentration effect. The nucleation rate of crystals depends on the degree of supersaturation [68]. Due to freeze concentration, the supersaturation is increased and consequently, the nucleation rate is increased as well. For pure NaCl systems, above 1.82 M mainly Cl VI and Cl VII appeared, indicating a shift to sea urchin crystals. It can be assumed that the initial viscosity in these formulations (due to temperature [69] and protein concentration) was very high leading to a lower diffusion rate, which in return results in a lower nucleation rate [70]. Next to the lower nucleation rate, high supersaturation, as well as temporary LLPS, is another prerequisite for the growth of sea urchin crystals [17, 66]. The application of FT stress to sucrose formulations resulted in an overall lower decrease in protein solubility. Thus, the known effectiveness of sucrose as a cryoprotectant could be confirmed with these observations [45, 46]. The dominating cluster formation of Cl V and Cl VI and spread towards lower excipient concentrations mimics the above stated line of argumentation, namely an increase in the nucleation rate due to freeze concentration [17, 62, 66, 68]. For glycerol containing systems, FT stress application resulted in a smaller occurrence of Cl I and a decrease in solubility, but less significant than for NaCl formulations, see Figs. 2b and 3b. Subsequently, glycerol was as expected able to stabilize lysozyme regarding FT stress. The intermediate cluster transition from small (Cl IV) to bigger (Cl II) crystals seen for FT c1 took place at protein concentrations below 2 mg/mL lysozyme. This might be an indication for a situation where FT stress is no yet the dominating factor and the addition of glycerol resulted in slower aggregation kinetics and larger crystals [35]. When performing the FT cycles three times the observed cluster formation of Cl VI and its positioning resembled the above-described dominant situation. PEG200 showed no stabilizing effect, seen in the decrease of Cl I, compared to the NaCl formulations. Overall, as found for the other systems, the scheme of a dominant growth of Cl IV is seen and due to the above-described combination of FT stress. The systems containing Tween20 already started with a dominating Cl V. With an increase in FT stress, a clear decrease in Cl I was observed pointing towards the inability of the current formulation to act as stabilizing formulation under the given conditions. This is underlined by the most dominant appearance of Cl IV during FT cycling over the whole aggregation zone.

Overall, the highest values of the calculated SL area were reached with the osmolytes tested (sucrose and glycerol) due to preferential hydration of the surface and minimized protein–protein interactions. The smaller the protein–protein interactions, the higher the solubility [23, 35]. Consequently, sucrose and glycerol are the excipients that were able to stabilize lysozyme the most regarding solubility and size of the solubility zone during FT cycling. PEG did not change stability significantly. For all systems investigated the addition of Tween20 led to destabilization when compared to pure NaCl containing systems, which is probably due to the applied concentration.

#### Heat cycle–FT c1 h and FT c3 h

When applying heat cycling after FT cycling a clear cluster transformation and thus reversibility of already formed aggregates resulting in a decrease of Cl IV and increase in Cl I as well as an increase in protein solubility (Fig. 3) was seen. This clearly shows that heat cycling might be applied to exploit the reversibility of aggregates formed not only during FT cycling but also during general processing, i.e. other unit operations creating aggregates. In general, mainly Cl IV areas transformed back to Cl I, but also Cl II and Cl III. In common to these clusters is either no *t*_onset_ for Cl I (fully soluble area) or *t*_onset_ > t_0_ for Cl II and Cl III, whereas the other clusters (Cl IV–Cl VII) showed aggregates from the beginning when the plates were pictured the first time *t*_onset_ = *t*_0_. The heat-induced reversibility is assumed to lead to a reset of the reversible systems and new arrangement of crystals independent of and undisturbed by stress resulting from FT or heat cycling.

A potential cause for a reset of systems – in this study mainly systems lying in the metastable zone containing reversible aggregates by heat cycling might be that the applied heat / energy input was high enough to loosen protein–protein interactions (only weak non–covalent protein interactions) [26], but also low enough not to induce heat aggregation and/or unfolding [28]. In this context, it is assumed that aggregates in the lower supersaturated region (adjacent to Cl I) created weak protein–protein interactions and, therefore, those are able to dissolve by heat [26], and consequently the same clusters are created than without FT stress or heat.

The good reversibility found for the osmolyte systems containing sucrose or glycerol is assumed to relate to weak protein–protein interactions [26, 69] and thus potentially reversible, due to the preferential hydration of the protein surface supported by the excipients.

Also for the PEG200 systems, heat-induced reversibility was observed. However, the effect was found not as significant as for NaCl formulations. On the contrary, PEG200 molecules seemed to stabilize the aggregates, which occurred due to the FT stress. The reason for this might be based on the overall higher polymer concentrations in solution due to freeze concentration, resulting in a displacement of the PEG molecules from between the protein molecules instead of steric stabilization [71]. Hence, the restructuring of the PEG molecules seems to be not completely reversible. For Tween20 formulations, the heat cycle only influences the occurrence of Cl I and the overall protein solubility. The protein solubility could be completely reversed by heat (Fig. 2b). The occurrence of Cl I, representing the soluble zone, was not completely reversed to the size observed at FT c0, but most of the FT stress-induced aggregates dissolved when heat was applied, see Fig. 2b.

In summary, the best performance (increase of Cl 1) in the heat-induced reversibility showed glycerol-containing systems. The follow-up systems were sucrose, pure NaCl and the Tween20 systems (the latter showing a reduced reversibility for increasing FT cycling). The lowest effect on heat-induced reversibility was seen for the PEG200 containing systems.

## Conclusion

In this study, the effects of instabilities induced by FT stress (up to three cycles) and the reversibility of those instabilities induced by heat cycling on the long-term (MPPD,SL) and short-term protein stability (size and structure) were investigated using lysozyme as an exemplary protein. It was shown that a re-set of areas consisting of reversible aggregates could be reached by heat cycling. This led to a resolution of reversible aggregates. The influence of different well-known cryoprotectants (sucrose, glycerol, PEG200 and Tween20) showed that the degree of instabilities and reversibility of aggregates was formulation dependent. The effects of FT-induced instabilities and their reversibility by heat-cycling, depending on the different formulations, can be summed up as follows: The addition of sucrose and glycerol resulted in the best performance as cryoprotectant. Regarding aggregate reversibility (increase in Cl I) glycerol performed best, followed by sucrose, NaCl and Tween. Finally, the use of MPPD to study the complexity and interplay of different formulations and processing situations showed to be excellent in terms of data visualization. In future work, the influence of heat cycling on reversible aggregation will be investigated. Furthermore, different formulation parameters like pH value, salts, cryoprotectants and buffer systems need to be investigated. The transfer to other protein molecules and also to highly concentrated protein formulations is mandatory for applications in industry. Finally, the addition of a heat cycle might be an effective tool to minimize instabilities throughout general processing.

## Electronic supplementary material

Below is the link to the electronic supplementary material.

Supplementary file1 (DOCX 411 kb)
