# Supplementary material for: Investigation of the reversibility of freeze/thaw stress-induced protein instability using heat cycling as a function of different cryoprotectants
Source: Bioprocess Biosyst Eng. 2020 Mar 20;43(7):1309–27. doi: 10.1007/s00449-020-02327-3 (PMC7261286; doi:10.1007/s00449-020-02327-3)
Supplement: Supplementary file 1 — Supplementary file1 (DOCX 411 kb) [file 449_2020_2327_MOESM1_ESM.docx]

**Supplementary Material**

**Investigation of the reversibility of freeze/thaw stress-induced instability using heat cycling as a function of different cryoprotectants**

Anna Katharina Wöll, Jürgen Hubbuch*

*Correspondence to Prof. Dr. Jürgen Hubbuch, telephone; +49 721 608 47526; fax: +49 721 608 46240; e-mail: [Juergen.hubbuch@kit.edu](mailto:Juergen.hubbuch@kit.edu)

1. MPPD construction
   1. Scatterplot

Figure S1 shows the position of all 2400 conditions regarding the RGB color code.


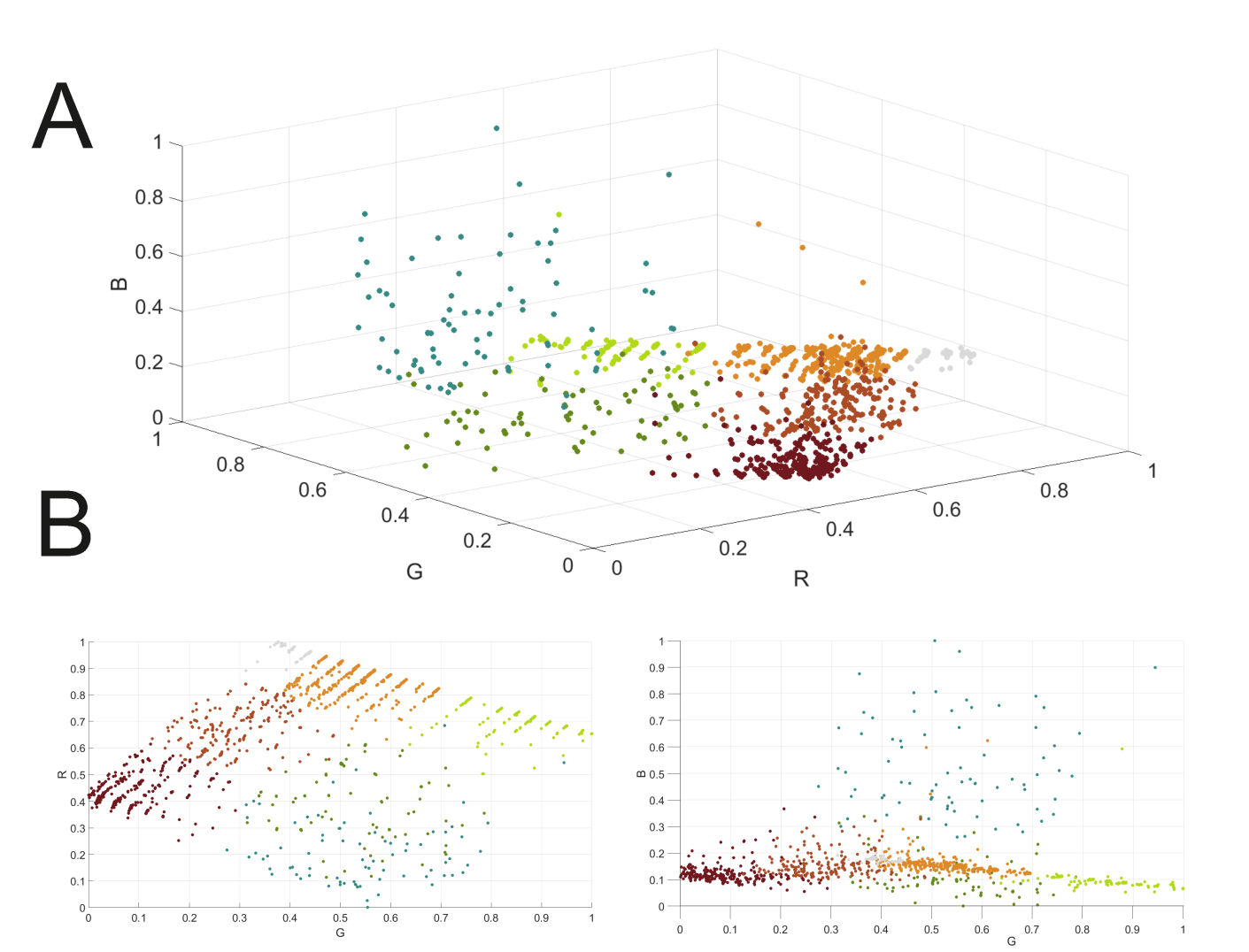


Figure S1: The position of all 2400 tested conditions regarding the RGB color code after clustering. in 3D (A) and 2D (B). Grey represents Cluster I. dark brown Cluster II. brown Cluster III. orange Cluster IV. green Cluster V. dark green Cluster VI. and blue Cluster VIII.

1. Analytic
   1. DLS data

The DLS data shown in Figure 4 in the manuscript are shown with the respective deviation from the triplicate measurements in Figure S2.


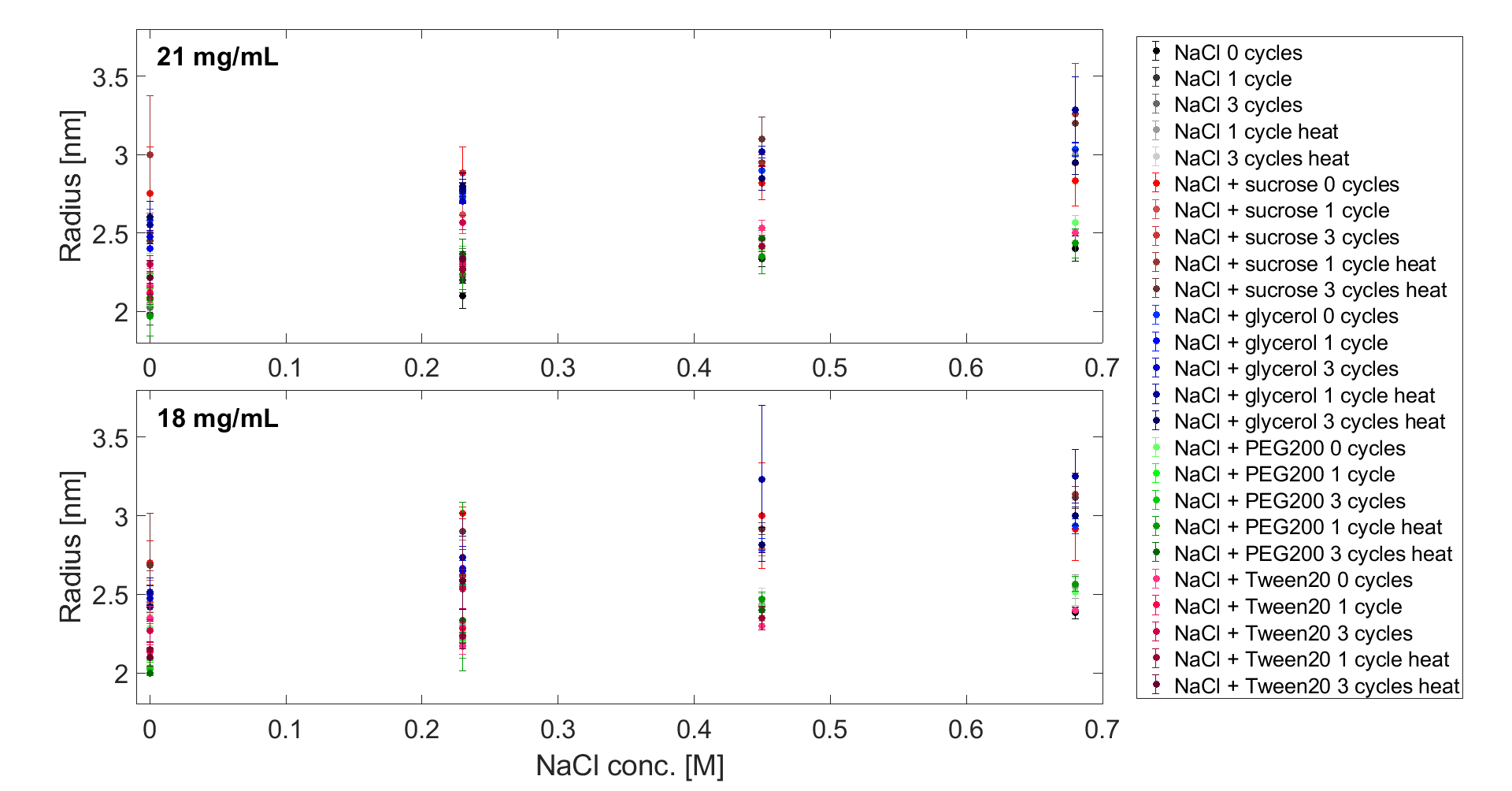


Figure S2: The DLS data plotted with the respective deviations for 21 mg/mL (top) and 18 mg/mL (bottom) lysozyme. The radius in nm is plotted over the NaCl concentration in M. All 153 conditions are plotted which performed different stress protocols.
